# Ultrahypofractionated radiotherapy for localised prostate cancer: The impact of daily MRI-guided adaptive radiotherapy on delivered dose

**DOI:** 10.1016/j.ctro.2025.100985

**Published:** 2025-06-03

**Authors:** S.E. Alexander, R.A. Mitchell, A. Dunlop, T. Herbert, K. Morrison, J. Nartey, U. Oelfke, H.A. McNair, A.C. Tree

**Affiliations:** a The Royal Marsden NHS Foundation Trust and the Institute of Cancer Research, UK; b The Joint Department of physics, the Royal Marsden Hospital and the Institute of Cancer Research, UK; c The Royal Marsden NHS Foundation Trust, UK

**Keywords:** Adaptive radiotherapy, MRI-guided radiotherapy, Ultrahypofractionated radiotherapy, Prostate cancer

## Abstract

**Introduction:**

Magnetic resonance image-guided adaptive radiotherapy (MRIgART) reduces uncertainties by correcting for day-to-day target and organ-at-risk deformation and motion. This is the first study to examine the dosimetric impact of MRIgART for ultrahypofractionated prostate cancer treatment, compared to standard-of-care image-guided non-adapted radiotherapy.

**Methods:**

Twenty patients with localised prostate cancer, who received ultrahypofractionated MRIgART on the Unity MR linac (Elekta, Sweden) were retrospectively analysed. Online daily MRI was acquired for replanning (MRI_session_) and a second for position verification before treatment (MRI_verification_). To compare delivered dose with and without adaptation, three plans were generated offline per fraction; a session plan (reference plan adapted to MRI_session_ anatomy), a verification plan (session plan recalculated on MRI_verfication_ anatomy), and a non-adapted plan (reference plan recalculated on MRI_verfication_ anatomy). Target and organ-at-risk doses were calculated, and dose difference evaluated.

Secondary analysis, using deformable dose accumulation, estimated verification and non-adapted dose to primary target (CTVpsv) substructures; prostate, gross tumour volume (GTV) and proximal 1 cm of seminal vesicles (1cmSV). Impact of prostate, rectum and bladder volume changes on dose were evaluated.

**Results:**

Median dose to 95 % of the CTVpsv was significantly higher with adaptation; 40.3, 40.0 and 38.2 Gy for session, verification, and non-adapted plans. Adaptation achieved a lower median urethra V42Gy dose but bladder V37Gy dose was lower when not adapting. Rectum V36Gy dose was similar for adapted and non-adapted plans.

CTVpsv substructure dose difference was greatest for 1cmSV; 40.0 versus 37.5 Gy for verification/non-adapted plans. Adaptation achieved significantly higher prostate only, but not GTV doses. Prostate and rectal volume changes had a negative impact on non-adapted dose only.

**Conclusion:**

MRIgART, offers significant dosimetric benefit for ultrahypofractionated prostate cancer compared to non-adapted strategies. Greatest benefit is expected for those with SV or high-risk of SV involvement, persistent rectal gas, prostate swelling and for the application of novel dose strategies including GTV dose escalation and non-involved prostate dose de-escalation.

## Highlights

• MRI-guided adaptation offers significant dosimetric benefit for ultrahypofractionated prostate cancer RT.
• Dosimetric benefits of MRIgART, compared to non-adapted strategies, are most apparent for target and urethra doses.
• MRIgART achieved significantly higher prostate and significantly lower urethra doses.
• Greatest benefit expected for individuals with; SV/high-risk of SV involvement, persistent rectal gas, prostate swelling and for the application of novel dose strategies.
• Prostate and rectal volume changes had a negative impact on non-adapted dose only.

## Introduction

Technical advances in radiotherapy planning and delivery have significantly improved prostate cancer outcomes. For example, the introduction of daily image-guidance, now standard of care [1], has been shown to reduce radiotherapy toxicity rates [[2], [3], [4]], improve patient outcomes [2,3] and facilitate safe dose escalation [5].

The advent of magnetic resonance image-guided adaptive radiotherapy (MRIgART) provides further opportunity to reduce uncertainties during prostate cancer radiotherapy, by correcting for interfraction target and organ-at-risk (OAR) deformation and motion [[6], [7], [8], [9], [10], [11]]. However, the consequence of these advanced features is lengthier more resource-intensive radiotherapy treatments [12,13].

To justify these consequences MRIgART must be shown to offer benefits beyond standard-of-care daily image-guided radiotherapy (IGRT) techniques. Adaptive radiotherapy has already been shown to achieve higher target doses [14,15] but has not been examined in ultrahypofractionated MRIgART for prostate cancer. Fewer radiotherapy fractions allow less scope for dose deviations to average out over treatment. This warrants our efforts to examine if MRIgART offers dosimetric benefits over IGRT, for this patient group, on a population and individual level.

## Materials and methods

We retrospectively analysed the datasets of 20 patients diagnosed with localised prostate cancer, who received MRIgART on the Unity MR linac (MRL) (Elekta, Stokholm, Sweden). Patients were prescribed 36.25 Gy in 5-fractions with a CTV to PTV margin of 5 mm except 3 mm posteriorly [16]. All were recruited to PERMIT and MOMENTUM, consenting to their images being used for research [17,18].

### Pre-treatment

Patients underwent a computer tomography (CT) planning scan in the supine head-first position, acquired at 1.5 mm slice intervals on a Siemens Confidence (Munich, Germany) scanner. Patient preparation included rectal emptying and a comfortably full bladder; micro enemas were used for two days prior to and 1–2 h before the planning CT and patients drank 350 ml of water 45-minutes prior to CT. Immobilisation comprised indexed knee and ankle support utilising the combifix^TM^ system (CQ Medical, Iowa, USA), head support with arms crossed on chest. A T2-weighted MRI, in the same position, was acquired on a 1.5 T Siemens Aera (Munich, Germany MRI scanner), and fused with the planning CT in RayStation (RaySearch, Stokholm, Sweden, 2023B V14.0.0.3338) to support target delineation.

Reference plans were generated in Monaco (Elekta, Stockholm, Sweden, V5.40.00). Key target and organ at risk (OAR) structures and their dosimetric constraints, aligned with the PACE C trial guidelines [16], are presented in Table 1. Target coverage was compromised where necessary to meet mandatory OAR dose constraints.

**Table 1:** Summary of key clinical structures and goals. Mandatory targets in brackets.

**Name**	**Structure**	**Dosimetric criteria**
CTVpsv_40.00	Prostate plus proximal 1 cm of seminal vesicles	V40.00 Gy > 95 %
PTVpsv_36.25	CTVpsv_4000 plus a 5 mm margin except posteriorly 3 mm	D0.1 cm^3^ < 48.33 Gy
		V36.25 Gy > 95 %
		D98% > 34.40 Gy
PTVsv_30.00	CTVpsv_4000 plus a 6 mm margin	V30.00 Gy > 95 %
Rectum	Includes the lumen and rectal wall, extending from (and including) the anus to the rectosigmoid junction	V36.00 Gy < 1 cm^3^ (2 cm^3^)
		V29.00 Gy < 20 %
		V18.10 Gy < 50 %
Bladder	Includes the bladder wall and lumen	V37.00 Gy < 5 cm^3^ (10 cm^3^)
		V18.10 Gy < 40 %
Bowel	Individual bowel loops extending from recto-sigmoid junction to 2 cm superior to CTVpsv	V30.00 Gy < 1 cm^3^
		V18.10 Gy < 5 cm^3^
Urethra	The lumen/mucosal interface extending from bladder neck to membranous urethra	V42.00 Gy < 50 %
Penile bulb	Portion of the bulbous spongiosum that lies inferior to the urogenital diaphragm	V29.50 Gy < 50 %
Femoral heads	From most cranial aspect to the bottom of the curvature of the femoral head	V14.50 Gy < 5 %

### Online clinical workflow

Patient preparation and immobilisation was as per the planning CT, with bladder filling reduced to 30-minutes to compensate for longer on-couch time.

Patients were treated following an MRI-guided adapt to shape (ATS) workflow [19,20]. A T2-weighted 3D transverse MRI, labelled MRI_session_, was acquired for recontouring and replanning. Online contours were reviewed and edited by a clinical oncologist and the reference plan optimised on the MRI_session_ by an MRL medical physicist. Plan parameters were modified, and target coverage compromised to meet mandatory OAR dose constraints, as necessary.

A second T2-weighted 3D transverse MRI was acquired at the latter stages of plan optimisation, labelled MRI_verification_. Prostate motion relative to the MRI_session_ was assessed and an adapt-to-position (ATP) workflow of the ATS plan [20,21] implemented to correct deviation, if the prostate had moved 3 mm or more in any cardinal direction.

Radiotherapy was delivered using 11-field intensity modulated radiotherapy (IMRT).

### Offline study workflow

Online adequate OAR volumes were acceptable but offline, to increase the accuracy of dosimetric analysis, OAR contours on the MRI_session_ were refined to be anatomically accurate. The bladder and rectum were delineated in full, plus bowel up to 3 cm cranial of the superior aspect of the prostate. The external body, femoral head contours and any other online delineation inaccuracies were also refined. Updated MRI_session_ anatomy was then propagated to the MRI_verification_ for each fraction. Propagation of the prostate, seminal vesicles, urethra, and penile bulb was rigid, for other structures deformable. MRI_verification_ contours were edited as required to accurately represent anatomy. All contours were reviewed and approved by an experienced MRL therapeutic radiographer [22].

**Fig. 1:**
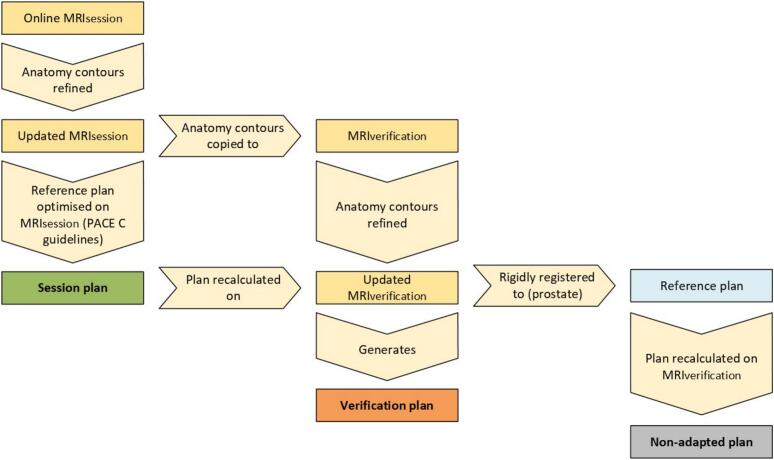
Overview of plan generation process.

The reference CT plan was optimised on each updated MRI_session_. Plan parameters were modified, as per online procedures, verified by an MRL medical physicist. These plans were termed the “session plan” for each fraction (Fig. 1).

The session plan was recalculated, but not adapted, on the contoured MRI_verification_, the steps taken to generate the “verification plan” (Fig. 1) varied depending on the need for ATP of ATS or not.

To mimic a standard non-adapted daily IGRT protocol the contoured MRI_verification_ was rigidly registered to the prostate on the reference CT. The whole prostate was considered during registration, however the superior and posterior borders, where the prostate abuts the bladder and rectum respectively, drove the match as they were the most reliably visualised on CT and MRI. The reference plan was then recalculated on the MRI_verification_ to generate the “non-adapted plan” (Fig. 1).

### Data analysis

Dosimetric and organ volume (prostate, bladder, and rectum) data from Monaco was evaluated on a group and individual level. PRISM (GraphPad, USA) was used to evaluate data normality, dose difference and correlation. For each plan type, average target and key OAR doses were calculated and Wilcoxon signed-rank tests performed for significance between the session and verification, verification and non-adapted and session and non-adapted plans. A significance level < 0.01 was set to account for multiple comparisons.

### CTVpsv sub-structure analysis

A secondary analysis was performed to estimate cumulative dose to the CTVpsv and its substructures (prostate, gross tumour volume (GTV) and proximal 1 cm of seminal vesicles (1cmSV)). For this MRI_verification_ images, updated structure sets, verification and non-adapted plans for all fractions were exported to research RayStation (RaySearch, Stokholm, Sweden, 2023BR V14.1.100).

For each patient, the dominant GTV was contoured on fraction one, guided by diagnostic MRI. The GTV was rigidly propagated to fractions 2–5 and translation and rotation corrections applied to each to position the GTV accurately. Manual editing of the GTV was permitted as necessitated by prostate deformation.

Deformable image registration between fraction one and all other fractions was then performed per patient, with the prostate and CTVpsv identified as controlling regions of interest. Fraction 2–5 doses were warped onto the fraction one image set using deformable registration. The five fractional doses for verification and non-adapted plans were then summed onto the fraction one image set to estimate cumulative dose for each plan strategy [23].

For the verification and non-adapted plans, correlation between CTVpsv sub-structure doses (per-fraction) and prostate, bladder and rectal volume and change in prostate, bladder and rectal volume from reference CT were examined. Spearman’s correlation (rs) was used with values of ≥ 0.5 and ≥ 0.7 set for moderate and high correlation respectively [24].

## Results

All patients completed five fractions of radiotherapy. ATP of ATS was used for 44/100 fractions, due to prostate motion seen on MRI_verification_. Median (range) time from MRI_session_ to MRI_verification_ was 30 (19–45) minutes.

**Fig. 2:**
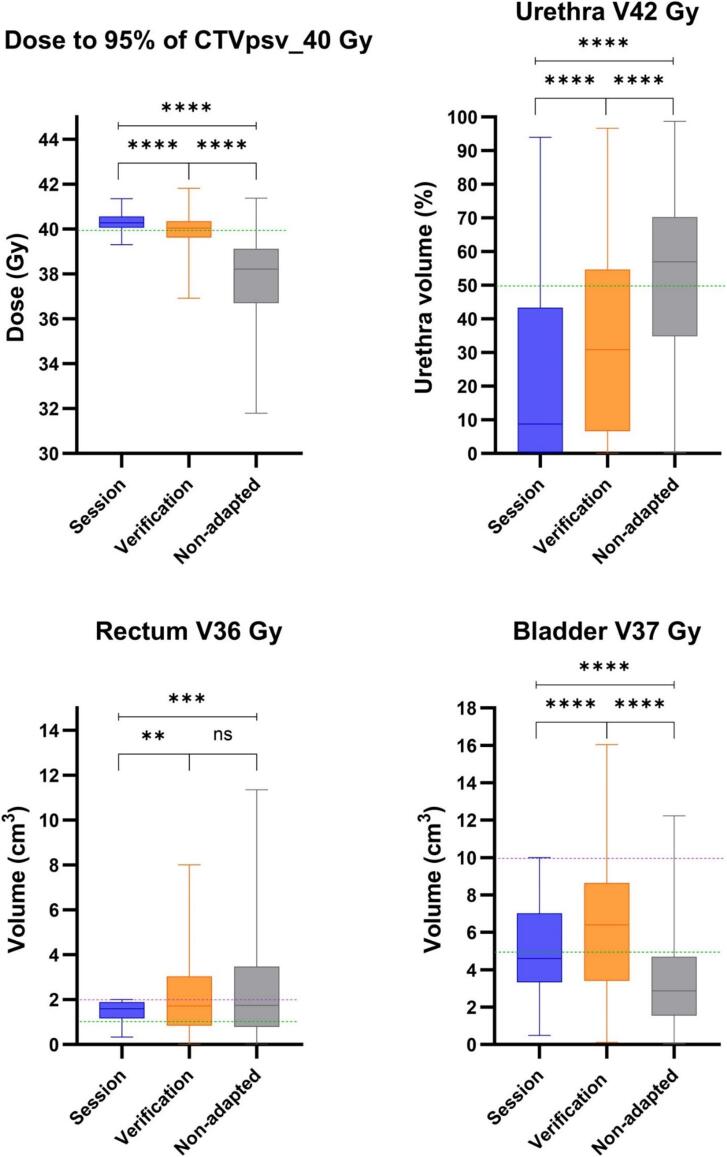

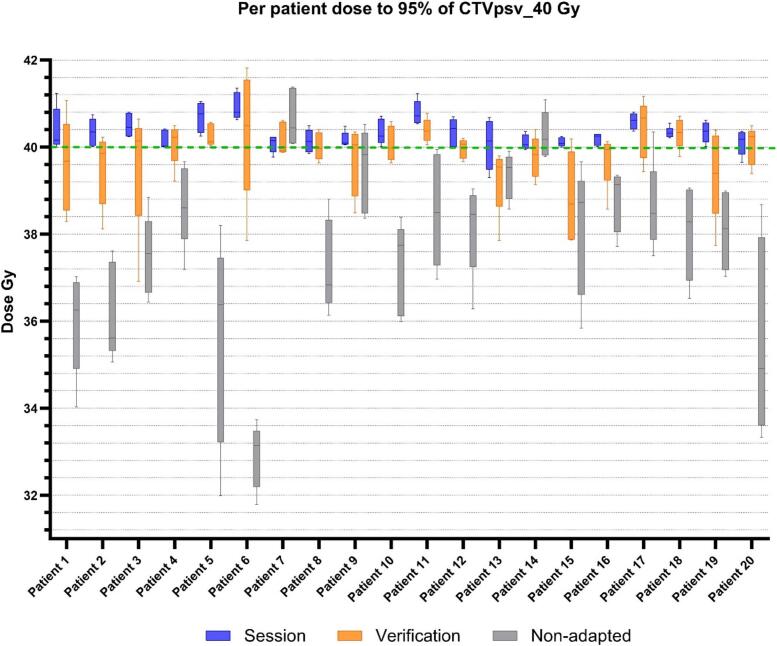
A (top): Median, interquartile and range dose to key target and OAR constraints. ns = not significant, ** = p < 0.01, *** = p < 0.001, **** = p < 0.0001. Green dotted line = optimal dose constraint, purple dotted line = mandatory dose constraint. Fig. 2B (bottom): Median dose to CTVpsv_40. Error bars represent the minimum and maximum daily plan doses, as though each plan delivered the entire treatment course. (For interpretation of the references to colour in this figure legend, the reader is referred to the web version of this article.). (For interpretation of the references to colour in this figure legend, the reader is referred to the web version of this article.)

Median (range) dose across all fractions for all patients, as though each plan delivered the entire treatment course, is presented in Fig. 2A for key target and OAR constraints. Median dose to 95 % of the CTVpsv_40.00 was 40.3, 40.0 and 38.2 Gy for the session, verification, and non-adapted plans, respectively. Urethra V42 Gy was significantly lower for the adapted plans; 8.8, 30.9 and 56.9 percent for the session, verification, and non-adapted plans. Conversely the bladder V37 Gy constraint was lowest for the non-adapted plans; 4.6, 6.4 and 2.9 cm^3^ for session, verification, and non-adapted plans. Less difference was seen in rectum V36 Gy between adapted and non-adapted plans; 1.6, 1.7 and 1.7 cm^3^ for session, verification, and non-adapted plans.

On a per fraction basis 95 % of the CTVpsv received 40 Gy or more for 93 % of session plan fractions, 55 % of verification plan fractions and 11 % of non-adapted fractions. Supplementary Material A presents the proportion of fractions meeting this and other key clinical goals for each planning strategy.

On a patient specific level, Fig. 2B presents median (range) dose across all fractions per patient, as though each plan delivered the entire treatment course. The session plan achieved the CTVpsv_40.00 constraint for all patients, the verification for 11/20, and the non-adapted plan for 2/20 patients.

**Fig. 3:**
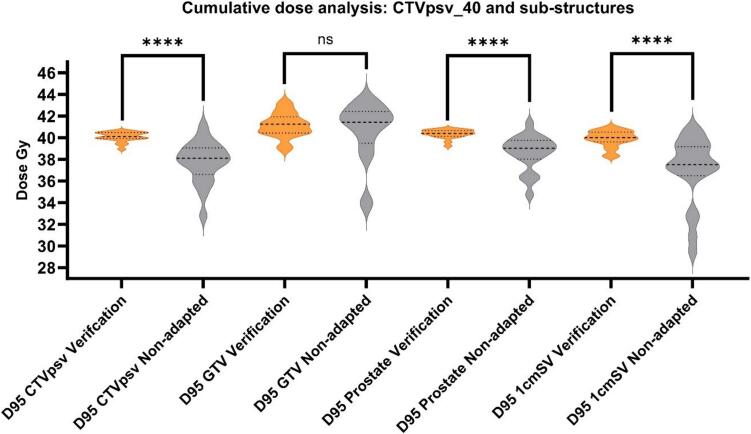
Group estimated cumulative dose to the CTVpsv and its substructures. Orange plots = verification plans, grey plots = non-adapted plans. ns = not significant, **** = p < 0.0001. (For interpretation of the references to colour in this figure legend, the reader is referred to the web version of this article.). (For interpretation of the references to colour in this figure legend, the reader is referred to the web version of this article.)

Group estimated cumulative dose to the CTVpsv and its substructures is presented in Fig. 3. Median (IQR) CTVpsv_40 dose was 40.1 Gy (39.9–40.5) and 38.1 Gy (37.1 – 39) for the verification and non-adapted plans, respectively. The verification and non-adapted plan difference for prostate only (removing proximal 1 cm of SV from the CTVpsv_40) was slightly less, 40.4 Gy (40.2–40.6) versus 39.0 Gy (38.1–39.6), with the greatest difference seen for 1cmSV, 40.0 Gy (39.7–40.5) versus 37.5 Gy (36.8–39.1). The difference in GTV coverage was not significant, 41.1 Gy (40.4–41.8) versus 41.4 Gy (39.3–42.2). Individual patient’s doses are presented in Supplementary MaterialB.

**Fig. 4:**
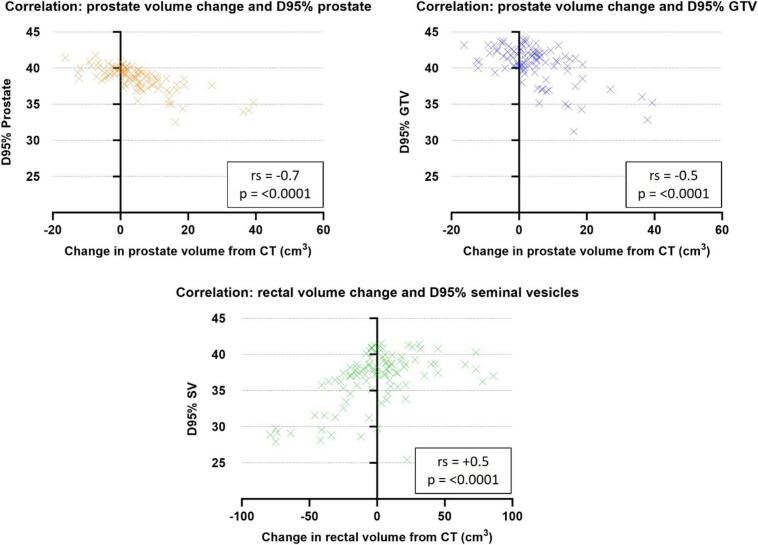
The only high and moderate Spearman’s correlation results were found for non-adapted plan CTVpsv substructure doses and prostate and rectum volume changes. rs ≥ 0.5 = moderate correlation, rs ≥ 0.7 = high correlation.

**Fig. 5:**
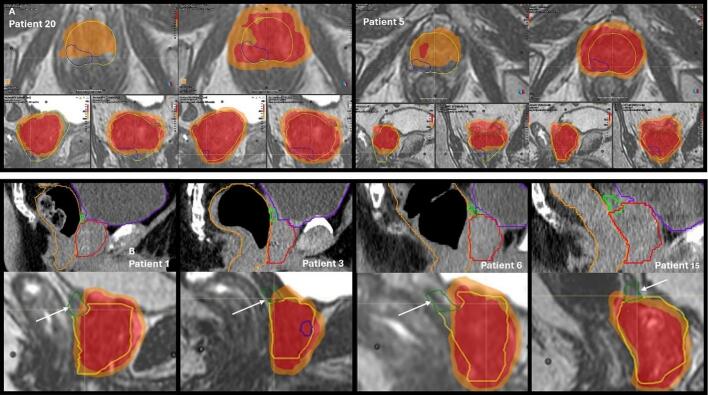
A shows patient 20 and 5, the non-adapted plan for #4 is on the left and the verification plan for #4 the right, red isodose = 40 Gy, orange isodose = 36.25 Gy. Cumulative prostate (yellow contour) and GTV (blue contour) dose dropped below 36.25 Gy on the non-adapted plan for both. B a sagittal view of the planning scan for patients 1, 3, 6 and 15 (top) and from #5 of their non-adapted plans (bottom), the white arrow indicates missed SVs (green contour) in the absence of adaptation. (For interpretation of the references to colour in this figure legend, the reader is referred to the web version of this article.). (For interpretation of the references to colour in this figure legend, the reader is referred to the web version of this article.)

Bladder, rectum, and prostate volume correlation with non-adapted and verification CTVpsv sub-structure dose is presented in Supplementary MaterialC, moderate to high correlation was only seen in the non-adapted plans (Fig. 4). Prostate (only) dose was significantly lower with non-adapted radiotherapy. Dose decrease was strongly correlated with an increase in prostate volume from planned i.e. greater prostate swelling during RT led to worse D95% coverage of the prostate (Fig. 4). Dose fall off was noted most at the inferior extent of the gland (Fig. 5A), for two patients D95% prostate dropped below 36.25 Gy. Correlation between prostate volume changes and dose was not seen in the verification plans, as swelling deformation was corrected.

Moderate negative correlation between D95% GTV and prostate volume increase was found with non-adapted radiotherapy (Fig. 4). Despite group GTV dose difference being insignificant, GTV dose did drop below 40 Gy and was ≥ 1.7 Gy lower with non-adaptive radiotherapy for 4/20 patients, these individuals all had a GTV volume in the posterior-lateral peripheral zone, mid-gland to apex. GTV dose dropped below 36.25 Gy for two of these patients (Fig. 5A).

Non-adapted D95% 1cmSV was moderately correlated with diminished rectal volume from planned (Fig. 4). For four patients D95% 1cmSV dropped below 36.25 Gy. This was most notable for patients who had high volume rectal gas on their planning scan (Fig. 5B).

## Discussion

For ultrahypofractionated prostate cancer radiotherapy, MRIgART delivered statistically higher doses to the CTVpsv than a simulated daily IGRT non-adapted workflow. Prior studies also report higher target doses with adaptive RT [14,15] although ours is the first to show the impact of adaptation in a patient cohort treated with ultrahypofractionated MRIgART. Organ motion and deformation between MRI_session_ and MRI_verification_ had a dosimetric impact on both target and organs at risk, as previously seen [25]. We have since tightened our motion threshold for ATP correction on the MRI_verification_ to 2 mm, in a bid to reduce the dosimetric impact of a lengthier workflow.

The impact on dosimetry of non-adapted was starker than that of adapted RT digressions over time. Clinical outcomes following ultrahypofractionated prostate radiotherapy, using non-adaptive radiotherapy are excellent with 5-year failure-free survival of 84–96 % and low rates of grade two or worse gastrointestinal and genitourinary toxicity [26,27]. Outcomes indicate that the dose received to the whole prostate, using non-adaptive radiotherapy, is sufficient for intermediate-risk prostate cancer. Therefore, inadvertent dose escalation, as achieved with MRIgART, may not increase clinical benefit and risks increased toxicity.

These results lead us to consider whole gland dose de-escalation when utilising MRIgART, as previous studies showing excellent biochemical control [26,27] would have delivered less than the intended prescription doses to the whole prostate.

In light of data showing the benefit of focal dose escalation to the MRI-visible tumour, personalised de-escalation is also considered. The DESTINATION (NCT05709496) study is currently assessing the feasibility of whole-prostate dose de-escalation using MRIgART. Unlike our patient cohort, DESTINATION patients have a simultaneous integrated boost to their GTV + 4 mm [28]. Our study found no significant difference in cumulative GTV dose when using adapted or non-adapted radiotherapy, meaning in the absence of a planned GTV boost MRI guided adaptive whole-prostate dose de-escalation could in fact risk underdosing the dominant intraprostatic lesion. This would be clinically unfavourable as if cancer does recur in the prostate, it is almost always at the site of dominant disease identified on MRI at diagnosis [29]. Delineating the GTV and applying specific planning objectives would however mitigate this risk.

Our results support MRIgART as a means of achieving a higher CTV dose, this could be particularly relevant for individuals with high-risk prostate cancer, where higher radiation doses may improve local control. GTV dose escalation to 45 Gy or beyond has been investigated for high-risk prostate cancers, with acceptable toxicity rates [30,31]. However, in these trials the median GTV dose, delivered with non-adapted RT, did not reach the intended dose due to OAR constraints [30,31]. MRIgART offers opportunity to optimise GTV dose delivered per fraction, to achieve planned dose [32,33].

Our data suggests MRIgART offers greatest benefit to those with a GTV situated in the posterior peripheral zone of the prostate, mid gland to apex. In the absence of adaptation these GTV volumes received lower doses, impacted by the soft tissue registration method employed. The prostate-bladder interface guided superior/inferior alignment, enabling maintenance of bladder tolerance doses without adaptation, seen previously [21]. However, this tactic meant in the presence of cranio-caudal prostate swelling, the greatest tissue deviation befell the prostate apex [10]. Non-adapted GTV dose was negatively correlated with prostate swelling, with greatest impact seen for the most inferior dominant lesions.

Prostate volume increase during ultrahypofractionated radiotherapy is significant [[8], [9], [10], [11]]. We found volume changes from planned to have the greatest impact on prostate dose, with underdosing most apparent at the caudal extent of the gland, where dimension changes have been reported to be greatest [10]. Predictors of prostate swelling are not agreed in the literature; baseline prostate size was not a significant contributor but a trend for larger prostates to swell more was seen [9,10]. The impact of androgen deprivation therapy on swelling is also contested [10,11], with too few numbers to draw firm conclusions. Further investigation of this phenomenon, on a larger scale, is warranted as if we could predict those at greatest risk of swelling upfront, they would form ideal MRIgART candidates.

Lower urethra dose, as found with MRIgART, would be expected to reduce treatment related toxicity. Dose to the urethra has been associated with the development of genitourinary toxicity in patients undergoing ultrahypofractionated radiotherapy for prostate cancer [34,35], however we acknowledge that genitourinary toxicity is multifactorial and does not depend on bladder and urethra dose alone. Efforts to optimise and reduce urethra dose for prostate cancer RT have been limited by poor visualisation of the anatomy on CT [36]. Only MRIgART platforms enable urethra visualisation adequate for online delineation, without the need for patient catheterisation.

The proximal 1cmSV suffered the greatest dose drop with non-adapted radiotherapy. This is not surprising as seminal vesicle motion is variable and distinct from prostate motion [[36], [37], [38], [39]] resulting in greater dosimetric impact from organ rotation and deformation [11,40]. The 1cmSV dose drop was most marked for individuals with high volume rectal gas at their planning scan, and smaller rectal volumes during RT. SV dose was not correlated with daily rectal volume, signifying that rectal volume consistency with planning, rather than absolute rectal volume impacted non-adapted dose coverage.

Rectal volume variability is dependent on the individual, their anatomy, and environmental factors [41,42], occurring irrespective of the intensity of bowel preparation given [[43], [44], [45], [46]]. Our results show MRIgART copes better with rectal volume fluctuations from planning, especially those caused by high volume gas which is more transient than stool. Previous publications have also cited that those with unfavourable anatomy at the time of reference planning benefit most from MRIgART [21]. Persistent large volume rectal gas at planning could therefore be considered a stratification factor for MRIgART, to improve SV dosimetry. Especially important for individuals with T3b disease or high risk of SV involvement [47,48], where under-treating comes with a higher risk of biochemical recurrence and worse overall survival [49,50].

The authors recognise limitations of this work. The restricted sample size of 20 patients could be considered a shortcoming. A sample size of 20 was justified based on two factors; several published MRIgART studies report on cohort sizes of this magnitude or less [15,21,25], and the volume of recontouring and re-planning required was feasible for the research team to complete. Calculating dose on the MRI_verification_ does not account for the impact of motion or deformation during treatment delivery. However, it was justified for this project because the timing of its acquisition is most akin to that of a pre-treatment cone beam CT, acquired for non-adapted radiotherapy. We are also reassured that prostate motion during treatment delivery is typically modest [[47], [51]] and can now be accounted for by Comprehensive Motion Monitoring (CMM) where motion is tracked with 3D cine MRI during beam on and the dose can be shifted to account for movement of the prostate.

There are also limitations to the average and deformable dose accumulation methods employed to estimate delivered dose in this project. The average fractional dose approach has been presented in several publications [21,31] however it does not use all information available in the data, limiting output precision. We also acknowledge that dose accumulation does not account for what is happening biologically, however it does function as a useful surrogate to see how well we are delivering physical dose to the target volumes over the entire course of treatment. Uncertainties associated with deformable dose accumulation are reported [23,[52], [53], [54]], although encouragingly prostate cancer is a site where dose propagation is used clinically [52]. We limited uncertainties associated with this approach by evaluating CTVpsv dose only, not OAR structures prone to greater deformation, and by using the manually contoured prostate and CTVpsv structures as controlling regions of interest.

## Conclusion

We conclude that the ability to modify treatment to day-to-day anatomical changes using MRIgART, offers significant dosimetric benefit for ultrahypofractionated prostate cancer treatment. These dosimetric benefits, compared to non-adapted strategies, are most apparent for target and urethra doses. Greatest MRIgART benefit is expected to be for individuals with T3b disease or high risk of SV involvement, those where dose to the GTV is optimised and dose de-escalation to the non-involved prostate is used, persons with persistent high volume rectal gas at planning and those at greater risk of prostate swelling. Implementation of these stratification factors to streamline prostate cancer patient referrals for MRIgART has potential to optimise the clinical benefit of this contemporary technique.

Further work is planned to establish if predictive factors for prostate swelling exist and to validate the extra benefit MRIgART offers individuals meeting stratification factors identified. In the meantime, the results amplify our confidence in MRIgART to deliver ultrahypofractionated prostate cancer radiotherapy prescriptions as planned and allows us to consider novel dose strategies to optimise patient outcome.

## Patient consent statement

Data used was from participants recruited to the following trials who consented to their images being used for research.

• PERMIT (NCT03727698).
• MOMENTUM (NCT04075305).

## Funding statement

No specific funding for this project.

## Data availability statement for this work

Research data are not available at this time.

## CRediT authorship contribution statement

**S.E. Alexander:** Conceptualization, Data curation, Formal analysis, Investigation, Methodology, Project administration, Validation, Writing – original draft. **R.A. Mitchell:** Conceptualization, Data curation, Formal analysis, Methodology, Writing – review & editing. **A. Dunlop:** Formal analysis, Methodology, Writing – review & editing. **T. Herbert:** Project administration, Writing – review & editing. **K. Morrison:** Investigation, Project administration, Writing – review & editing. **J. Nartey:** Investigation, Project administration, Writing – review & editing. **U. Oelfke:** Supervision, Writing – review & editing. **H.A. McNair:** Methodology, Supervision, Writing – review & editing. **A.C. Tree:** Methodology, Supervision, Writing – review & editing.

## Declaration of competing interest

The authors declare the following financial interests/personal relationships which may be considered as potential competing interests: *S.E. Alexander:* Cancer Research UK Programme Grant C33589/A28284. *R.A. Mitchell:* Cancer Research UK Programme Grant. Elekta Research agreement. *A. Dunlop:* NIHR Senior Clinical and Practitioner Research Award (SCPRA) holder. *U. Oelfke*: CRUK Program Grant, Adaptive Data-Driven Radiation Oncology, C33589/A28284, see ICMJE Disclosure form for complete list. *H.A. McNair:* Royal Marsden Cancer charity. *A.C. Tree:* Institution research funding from Elekta, Accuracy & Varian, see ICMJE Disclosure form for complete list.
