# Supplementary material for: Ultrahypofractionated radiotherapy for localised prostate cancer: The impact of daily MRI-guided adaptive radiotherapy on delivered dose
Source: Clin Transl Radiat Oncol. 2025 Jun 3;53:100985. doi: 10.1016/j.ctro.2025.100985 (PMC12173759; doi:10.1016/j.ctro.2025.100985)
Supplement: Supplementary Data 1 [file mmc1.docx]

**Supplementary material A:** Proportion of fractions where clinical goals are met for each plan group

| Region of interest | Metric | Mandatory clinical goal (unless optimal (opt) stated) | MRL session plan | | MRL verification plan | | Non-adapted plan | |
| --- | --- | --- | --- | --- | --- | --- | --- | --- |
|  |  |  | **Median (IQR)** | **% fractions meeting goal** | **Median (IQR)** | **% fractions meeting goal** | **Median (IQR)** | **% fractions meeting goal** |
| CTVpsv_40 | **V40.00 Gy** | **≥ 95 %** | 96.9  (95.4–97.7) | 93 | 95.3  (93.2-96.6) | 55 | 86.7  (82.3-90.4) | 11 |
| Rectum | **V36.00 Gy** | **< 2 cm^3^**  **(opt < 1 cm^3^)** | 1.6  (1.2-1.9) | 100 (16) | 1.7  (0.9-3.0) | 55 (29) | 1.7  (0.8-3.5) | 55 (29) |
|  | **V29.00 Gy** | **< 20%** | 11.6  (9.1-13.6) | 100 | 11.7  (8.6-14.4) | 95 | 10.0  (7.6-14.2) | 93 |
|  | **V18.10 Gy** | **< 50%** | 37.4  (32.9-41.7) | 100 | 37.5  (31.1-72.7) | 98 | 33.9  (27.8-39.6) | 98 |
| Bladder | **V37.00 Gy** | **< 10 cm^3^**  **(opt < 5 cm^3^)** | 4.9  (3.6-7.6) | 95 (52) | 6.3  (3.3-8.8) | 84 (38) | 3.0  (1.8-4.9) | 92 (76) |
|  | **V18.10 Gy** | **< 50%** | 21.6  (14.0-29.6) | 99 | 13.5  (8.7-19.3) | 100 | 10.1  (7.1-18.3) | 99 |
| Urethra | **V42.00 Gy** | **opt < 50%** | 8.8  (0.4-43.1) | 81 | 30.9  (7.1-54.3) | 71 | 56.9  (36.5-69.9) | 36 |
| Penile bulb | **V29.50 Gy** | **opt < 50%** | 0.0  (0.0-0.0) | 100 | 0.0  (0.0-4.5) | 100 | 0.0  (0.0-0.0) | 100 |
| Bowel | **V30.00 Gy** | **< 1 cm^3^** | 0.0  (0.0-0.0) | 100 | 0.0  (0.0-0.0) | 98 | 0.0  (0.0-0.0) | 97 |
|  | **V18.10 Gy** | **< 5 cm^3^** | 0.0  (0.0-0.0) | 100 | 0.0  (0.0-0.0) | 98 | 0.0  (0.0-0.0) | 97 |

| **Patient** | **D95 CTVpsv_4000 Verification plan (Gy)** | **D95 CTVpsv_4000 Not-adapted plan (Gy)** | **D95 GTV Verification plan (Gy)** | **D95 GTV Not-adapted plan (Gy)** | **D95 Prostate Verification plan (Gy)** | **D95 Prostate Not-adapted plan (Gy)** | **D95 1cm SV Verification plan (Gy)** | **D95 1cm SV Not-adapted plan (Gy)** |
| --- | --- | --- | --- | --- | --- | --- | --- | --- |
| 1 | 40.0 | 35.9 | 39.0 | 39.5 | 40.6 | 39.8 | 39.7 | 30.9 |
| 2 | 39.5 | 36.4 | 43.0 | 41.4 | 39.6 | 36.5 | 40.1 | 37.5 |
| 3 | 39.9 | 37.3 | 40.7 | 41.9 | 40.3 | 39.2 | 39.9 | 33.1 |
| 4 | 40.6 | 39.1 | 40.5 | 40.6 | 40.6 | 39.2 | 41.0 | 39.8 |
| 5 | 40.4 | 34.8 | 39.2 | 33.7 | 40.6 | 34.8 | 40.8 | 39.0 |
| 6 | 40.7 | 32.8 | 43.3 | 43.2 | 40.8 | 39.8 | 40.7 | 29.5 |
| 7 | 40.4 | 41.1 | 41.4 | 42.1 | 40.8 | 41.4 | 39.6 | 39.8 |
| 8 | 40.3 | 37.6 | 42.4 | 38.2 | 40.4 | 38.0 | 39.7 | 37.1 |
| 9 | 39.9 | 39.6 | 41.3 | 41.4 | 40.1 | 39.6 | 40.3 | 40.8 |
| 10 | 40.0 | 37.3 | 40.6 | 40.4 | 40.1 | 36.5 | 39.9 | 38.3 |
| 11 | 40.5 | 38.7 | 41.7 | 41.6 | 40.8 | 38.9 | 40.3 | 39.2 |
| 12 | 40.4 | 38.1 | 41.0 | 41.4 | 40.5 | 38.6 | 40.7 | 37.0 |
| 13 | 39.9 | 39.3 | 40.3 | 42.4 | 40.4 | 40.2 | 39.2 | 37.8 |
| 14 | 40.2 | 40.2 | 41.4 | 43.7 | 40.4 | 40.3 | 40.5 | 39.1 |
| 15 | 39.0 | 37.8 | n/a | n/a | 39.3 | 39.2 | 38.9 | 32.4 |
| 16 | 40.0 | 38.9 | 41.3 | 42.2 | 40.3 | 39.4 | 40.0 | 37.0 |
| 17 | 40.6 | 38.1 | 42.4 | 43.0 | 40.8 | 38.1 | 40.1 | 40.7 |
| 18 | 40.5 | 38.2 | 40.4 | 38.7 | 40.7 | 38.6 | 40.5 | 37.6 |
| 19 | 39.4 | 38.2 | 41.9 | 42.8 | 40.1 | 38.7 | 38.3 | 36.3 |
| 20 | 40.1 | 35.8 | 40.4 | 34.3 | 40.4 | 36.1 | 38.4 | 37.4 |

**Supplementary material B:** Per patient cumulative doses to the CTVpsv and its sub structures for verification and not-adapted plans.

**Supplementary material C:** Bladder, rectum and prostate volume correlation with verification and non-adapted CTVpsv sub-structure dose. Significant *p*=<0.01. Moderate and high correlations marked with *.
